# Supplementary material for: Functional Yogurt Fortified with Honey Produced by Feeding Bees Natural Plant Extracts for Controlling Human Blood Sugar Level
Source: Plants (Basel). 2022 May 24;11(11):1391. doi: 10.3390/plants11111391 (PMC9182764; doi:10.3390/plants11111391)
Supplement: Supplementary file 1 [file plants-11-01391-s001.zip › plants-1717331-supplementary.pdf]

Table S1. Basic blood parameters of volunteers at the beginning and the end of clinical trial for each studied group

Measured paramters	Group (1)		Group (2)		Group (3)		Group (4)	
	Before	After	Before	After	Before	After	Before	After
Serum sugar touches (hungry)	4.77	4.27	4.91	4.92	5.28	5.07	4.81	3.73
HbA1c	5.35	------	5.52	5.45	5.62	5.20	5.29	4.90
HbA1c mmol / mol	35.3	-------	36.9	35.5	38.0	33.0	34.3	30.0
Urea touches. from serum	4.56	4.45	5.54	5.15	5.01	4.90	5.16	4.84
Determination of creatinine / enzymatic /	62.5	65.4	76.1	67.7	63.3	63.7	69.3	71.1
eGFR-EPI	89.3	90.0	82.8	87.4	88.1	90.0	87.8	88.3
Determination of sodium from serum	141	141	140	140	142	140	141	141
Potassium touches. In serum	4.54	4.36	4.48	4.58	4.61	4.42	4.36	4.19
Determination of chloride in serum	106	104	107	106	106	106	106	105
GOT me a decision	20.9	19.3	22.6	24.9	19.4	20.7	19.7	20.3
Definition of GPT	22.6	21.5	28.2	27.0	24.1	19.5	20.9	19.1
L-T-glutamyl transferase.	30.7	33.7	41.1	33.5	25.1	19.7	20.8	19.8
Determination of alkaline phosphatase	182	176	181	156	179	150	178	171
Determination of cholesterol (total)	5.3	5.3	6.0	6.0	5.3	5.2	5.4	5.4
Determination of HDL cholesterol	1.54	1.53	1.28	1.27	1.42	1.39	1.53	1.53
Determination of LDL cholesterol	3.56	3.51	3.86	3.88	3.21	2.99	3.33	3.24
Determination of triglycerides	1.25	1.15	2.63	3.45	1.99	1.65	1.10	1.14
Uric acid touches. from serum	303	273	323	312	288	312	260	265
Determination of cholinesterase	8523	8315	8744	8164	8454	7618	7824	7495
Determination of creatine kinase	138	123	149	170	145	164	129	140
Lactate dehydrogenase	448	389	481	448	392	462	421	422
Alpha-amylase affects. in serum	59.8	61.1	47.9	44.3	57.5	51.7	71.7	64.4
Determination of lipase	36.8	48.5	27.2	37.2	42.4	44.2	41.3	38.9
Calcium from serum	2.33	2.33	2.28	2.26	2.30	2.27	2.29	2.32
Determination of magnesium from serum	0.87	1.01	0.89	0.89	0.88	0.87	0.86	0.89
Determination of iron from serum	18.3	12.8	15.2	16.7	15.2	15.9	16.3	17.5
Determination of total iron-binding capacity	62.3	59.9	61.5	59.0	61.7	53.7	59.9	56.1
Transferrin	2.61	2.80	2.51	2.70	2.73	2.51	2.67	2.63
Determination of ferritin	71.4	62.6	136.5	183.0	93.7	108.9	77.5	84.6
Quantitative determination of reactive protein C (CRP)	1.97	8.90	2.76	1.52	2.39	0.36	1.71	0.37
Serum total protein	69.07	63.79	68.64	68.07	70.14	68.80	70.88	67.93
Determination of albumin in serum	45.33	44.64	43.64	43.36	43.79	44.10	46.13	45.36
Albumin	65.96	66.73	69.30	68.29	65.20	65.70	63.80	70.4
Alpha-1 globulin	2.27	2.30	2.15	2.14	3.00	2.53	2.70	2.70
Alpha-2 globulin	8.88	8.74	8.20	8.49	9.70	36.30	9.00	7.60
β-globulin	9.99	10.35	10.65	10.41	10.90	10.47	13.30	9.30
Gamma globulin	12.9	11.8	9.7	10.7	11.2	12.3	10.2	10.7
BRANCH	1.97	2.03	5.95	2.19	1.90	2.07	1.80	2.40
Immunoglobulin G	13.0	12.5	12.7	12.0	12.5	12.3	12.5	11.7

Immunoglobulin A	2.16	2.15	1.98	2.20	2.46	2.23	2.53	2.45
Immunoglobulin M	1.27	1.24	0.94	0.88	1.20	1.34	1.25	1.22
White blood cell count	7.33	7.03	8.17	7.10	7.34	7.17	7.00	6.84
Red blood cell count	4.66	4.75	4.60	4.79	4.69	4.58	4.82	31.41
Hemoglobin	143	138	141	140	135	135	138	136
Hematocrit	0.42	0.42	0.41	0.43	0.42	0.41	0.42	0.42
MCV	90.0	88.9	89.6	89.1	88.8	161.2	87.6	88.7
MCH	30.7	27.6	30.7	29.4	29.0	29.4	28.7	28.7
MCHC	340	329	342	324	326	329	328	318
Absolute neutrophil	4.00	3.91	4.96	4.16	4.31	4.22	4.25	4.15
Neutrophil	54.4	55.1	60.5	57.9	58.1	62.4	59.9	59.9
Absolute lymphocyte	2.56	2.33	2.37	2.10	2.30	1.94	1.98	1.91
Lymphocyte	35.0	33.5	28.9	31.8	31.6	27.7	28.9	28.6
Absolute monocyte	0.53	0.54	0.53	0.53	0.57	0.52	0.55	0.57
Monocyte	7.13	7.79	6.85	7.53	7.79	7.50	8.00	8.60
Absolute eosinophil	0.16	2.06	0.22	0.15	0.14	0.15	0.16	0.17
Eosinophil	2.33	2.29	2.85	2.47	2.00	2.40	2.25	2.56
Absolute basophil	0.06	0.05	0.04	0.04	0.04	0.05	0.05	0.04
Basophil	0.67	0.86	0.46	0.60	0.86	0.80	0.75	0.75
Prothrombin, Time	10.87	9.98	11.16	11.21	0.34	0.28	0.35	0.29
Activated partial thromboplastin time (Control)	29.7	30.9	29.2	30.6	1.0	1.0	11.0	10.9
Activated partial thromboplastin time (Patient)	30.7	28.2	30.4	28.8	30.1	29.6	1.0	1.0
Thrombin time (Control)	20.3	19.9	20.2	20.0	30.9	30.3	30.0	30.0
Thrombin time (Sick)	18.4	17.0	17.9	17.6	19.3	20.0	32.5	32.3

Table S2: List of the WHO code, and the methods, which used to measure all parameters in Table 2 and their instruments

WHO code	*Test Name*	**INSTRUMENT**	**METHOD**
21020	Determination of total protein in serum	Siemens Advia XPT	Biuret method without samples
21040	Determination of albumin in serum by the dye - binding method	Siemens Advia XPT	Bromocresol green (BCG) method
21072	Quantitative determination of reactive protein C (CRP)	Siemens Advia XPT	Immunoturbidimetry
21120	Determination of urea in serum	Siemens Advia XPT	Urease-GLDH method, enzymatic UV test
21130	Determination of uric acid	Siemens Advia XPT	Uricase / POD / 4-aminoantipyrine ADPS method with ascorbate oxidase (ASOD)
21143	Determination of creatinine by enzymatic method	Siemens Advia XPT	Enzymatic photometric test (PAP method)
21312	Determination of glucose by the hexokinase method	Siemens Advia XPT	Hexokinase method
21411	Determination of triglycerides	Siemens Advia XPT	GPO-PAP method
2142A	Determination of HDL cholesterol by direct method	Siemens Advia XPT	Direct HDL cholesterol method
21420	Determination of total cholesterol	Siemens Advia XPT	Enzymatic CHOD-PAP method
21422	Determination of LDL cholesterol by direct method	Siemens Advia XPT	Direct LDL cholesterol method
21500	Determination of sodium in serum	Siemens Advia XPT	Ion selective electrode (ISE), indirect potentiometry
21501	Determination of potassium in serum	Siemens Advia XPT	Ion selective electrode (ISE), indirect potentiometry
21510	Determination of total calcium	Siemens Advia XPT	Ortho-cresolphthalein complexone method
21571	Determination of magnesium	Siemens Advia XPT	Xylidyl blue color former
22885	Determination of alpha-amylase in body fluids	Siemens Advia XPT	EPS-G7 (liquid)
24500	Determination of lactic acid dehydrogenase (LDH)	Siemens Advia XPT	DGKCh method
24600	Determination of aspartate aminotransferase (ASAT, GOT)	Siemens Advia XPT	IFCC standardized method without pyridoxal phosphate
24610	Determination of alanine aminotransferase (ALAT, SGPT)	Siemens Advia XPT	IFCC standardized method without pyridoxal phosphate

24620	Determination of creatine kinase (CK)	Siemens Advia XPT	IFCC reference method
24640	Determination of gamma-glutamyltransferase	Siemens Advia XPT	IFCC reference method
24700	Determination of alpha-amylase in serum	Siemens Advia XPT	EPS-G7 (liquid)
24710	Determination of lipase	Siemens Advia XPT	Enzymatic photometric test (liquid)
24720	Determination of alkaline phosphatase	Siemens Advia XPT	Standard (DGKCh) method
24741	Determination of pseudo-cholinesterase	Siemens Advia XPT	Substrate: butyrylthiocholine iodide, DGKCh'94
2678A	Determination of IgM	Siemens Advia XPT	Turbidimetry
26780	Determination of IgG	Siemens Advia XPT	Turbidimetry
26788	Determination of IgA	Siemens Advia XPT	Turbidimetry
28014	Blood count with automatic machine IV.	Sysmex XN 1000	Machine counting of electrical / optical pulses
28330	Determination of iron-binding capacity	Siemens Advia XPT	ADVIA CHEMISTRY TIBC
28350	Definition of iron	Siemens Advia XPT	Ferrosin / Ferrene method
28360	Determination of total transferrin	Siemens Advia XPT	Turbidimetry
28390	Determination of ferritin	Siemens Advia XPT	Immunoturbidimetry
28494	Determination of hemoglobin A1c by HPLC, mass spectrometry	Arkray ADAMS HA-8180	Ion exchange chromatography
28610	Determination of thrombin time	Sysmex CS-5100	Turbidimetry
28620	Determination of prothrombin	Sysmex CS-5100	Turbidimetry
28621	Activated partial thromboplastin time	Sysmex CS-5100	Turbidimetry
